# Research on the identification and evolution of health industry policy instruments in China

**DOI:** 10.3389/fpubh.2024.1264827

**Published:** 2024-02-19

**Authors:** Jian Jin, Hongbin Du

**Affiliations:** School of Economics, Hebei University, Baoding, China

**Keywords:** health industry policy, policy instrument, BERT, deep learning, multi-label classification

## Abstract

The application of health industry policies could be discovered more quickly and comprehensively through the automated identification of policy tools, which could provide references for the formulation, implementation, and optimization of subsequent policies in each province. This study applies the Bidirectional Encoder Representation from Transformer (BERT) model to identify policy tools automatically, utilizes Focal Loss to reduce the unbalance of a dataset, and analyzes the evolution of policy tools in each province, which contains time, space, and topic. The research demonstrates that the BERT model can improve the accuracy of classification, that supply and environment policy tools are more prevalent than demand tools, and that policy instruments are organized similarly in four major economic regions. Moreover, the policy’s attention to topics related to healthcare, medicine, and pollution has gradually shifted to other topics, and the extent of policy attention continues to be concentrated on the health service industry, with less attention paid to the manufacturing industry from the keywords of the various topics.

## 1 Introduction

In recent years, people have paid more and more attention to health, and the scale of the Chinese health industry has been continuously expanding. The policies introduced by the government have provided powerful assistance to the development of the health industry in various aspects. Each province, autonomous region, and municipality have formulated a successive policy according to the characteristics of the region, especially after the State Council issued the “Healthy China 2030” planning outline, which has greatly promoted the development of the health industry in each province. Policy instruments mean the government applies policies into effect and are actual means that the government selects in formulating for implementing policies (1). The policy instruments are the representation of the policy text and are relevant to policy design and policymaking (2). Therefore, the rational selection of policy instruments will have a positive and long-term influence on the development of the health industry.

Textual data are steadily paying more attention to researchers due to the diversification of data formats, and text mining is becoming increasingly prevalent in text analysis for tasks including sentiment analysis, information retrieval, and intelligent push. Most textual data originate from search engines, social platforms, media reports, and policy text, and policy text is normative and distinctive. Moreover, text-as-data research is rising in political science (3). Policy text has an alternative paragraph marker format, which is more challenging to segment into separate paragraph statements and the length of the cut statements varies. Therefore, manual coding for policy tools is still the conventional research paradigm because policy instrument classification is distributed unevenly. Employing machine learning methods to classify policy instruments would improve the efficiency, due to highly costly manual labeling; however, the accuracy of classification methods for identifying unevenly distributed samples still needs to be ameliorated.

In this situation, this study aims to address the following questions (1): What is the more efficient method to identify the policy instruments from the health industry policy texts? (2) How can these classified policy instruments detect the change of different dimensions in the health industry? To answer these questions, this study utilizes the BERT deep learning algorithm to automatically quantify the policy text and aims to reduce the elevated labor costs. In addition, it applies the classification results to analyze the distribution and evolution of health industry policy instruments in time, space, and topic, and the latent dirichlet allocation (LDA) topic model is applied to detect the variation of industrial strategic initiatives. This framework for evolution and comparative analysis has significant policy implications.

## 2 Literature review

In 1964, the Western economist E. S. Kirschen classified the general type of policy instruments into 64 types, and subsequently, Rothwell and Zegveld (4, 5) elaborated three approaches to innovation policy in the context of reindustrialization, classifying policy instruments into supply, demand, and environmental types concerning the object of the policy or the focus of its operation. Afterward, based on existing theories of the effects of government action and the choice patterns of policymakers, McDonnell and Elmore (6) proposed a generalized framework for the analysis of policy instruments that translates essential policy goals into tangible action mechanisms and classified them into four types, which are mandates, inducements, capacity-building, and system-changing. Schneider and Ingram (7) proposed a set of policy analysis frameworks in 1990 to capture the behavioral attributes of policy content. These analytical frameworks for policy instruments that focus on behavioral characteristics are to be based on individual decisions and behaviors. As a result, based on the behavioral patterns of the target group, policy instruments are classified into five categories: authority, incentives, capacity-building, symbolic and hortatory, and learning. In 2003, Howlett and Ramesh (1) classified policy instruments into voluntary, coercive, and hybrid by focusing on the level of government provision of goods and services. In 2007, Hood and Margetts (8) summarized the basic resources that governments possess and classified them as information-based, authority-based, treasure-based, and organization-based, and these four attributes can then serve as the basis for policy instruments that play a role in detection and effects.

From the current point of view, the basis of the classification of policy instruments lies in coding, and manual coding is tedious and time-consuming (9). There have been many analytical studies based on the theory of policy instruments, and the main research paradigm has been studied by combining manual labeling of policy instruments with two or multiple-dimension labels and building an analytical framework for policy instruments. The number of policies might be a suitable measure, which can prescribe the current condition and goal; however, the sentences in the policy text could identify the policy measures and instruments (10). The provincial policy analysis should not be neglected due to local governments’ distinctive measures, which might provide more samples to compare the differences (11). Policy instruments have been advocated by governments to address the structural determinants of health (12), but it is difficult to extend the research objects through a case study. States in America adopt a mix of policy instruments to regulate competitive food sold in schools to ameliorate the nutrition environment of the school, and policy instrument analysis with manual coding provides the reference for the effectiveness of the various policy instrument mixes (13). In addition, policy instrument study can be analyzed by study cases that are selected purposely (14). However, some policy instruments might take more than one category, and it is hard to distinguish the category clearly but choose the category with strength according to past experience (15). In medical device policymaking, the United Kingdom’s active intervention with a variety of tools provides the environment of coordinative actions according to the expert interview, compared with the unbalanced German policy tools (16).

The quantity of policy releases accumulates over time, and in the era of big data, attempting to apply methods such as machine learning and deep learning can improve the efficiency of policy instrument classification, such as multi-classification (17) and multi-label learning (18). The application of a plain Bayesian algorithm for the classification of policy datasets shortens the distance between manual coding and computers and increases efficiency (19). Topic modeling could enhance the capacity of addressing larger sets of policy data, which might boost the efficiency of the policy study (20). A neural network and its improved methods are enabled to potentially boost the model accuracy in classification missions (21). The Transformer algorithm (22) was proposed in 2017 to provide a boost to text analysis, and later the BERT algorithm (23) improved it, which can significantly improve the accuracy of classification prediction. It is proved that the pre-training model could improve the efficiency of text classification in the policy content (24). Moreover, changing Chinese pre-training models could improve the accuracy of the multi-class model (25).

In summary, there is still space for ameliorating the method to identify and detect the policy instruments from the research on relevant studies. First, most research mainly applies manual coding and subsequently illustrates with dimensional combination, which generates higher labor costs or utilizes the smaller sample to analyze policy text. This approach is unable to compare the application of health policy from provincial and topic dimensions. Second, some studies apply multi-class models for policy instrument classification, but each policy statement could be relevant to multiple tools, and fewer studies apply the multi-label classification method in policy instrument identification. This study applies the BERT multi-label classification method with Focal Loss to identify the health industry policy instrument. Furthermore, the result of multi-label classification is utilized to discover the variation of health industry policy instruments in time, space, and topic dimensions.

## 3 Research design and methodology

**Figure 1:**
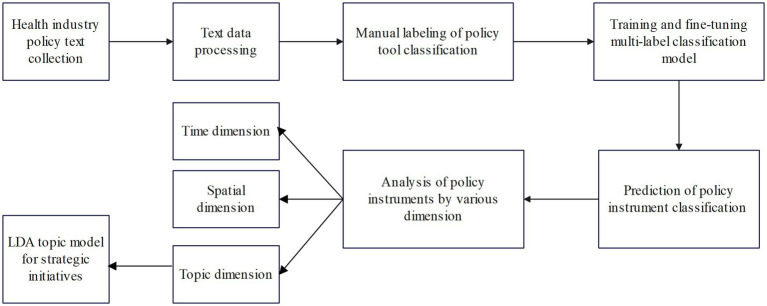
Policy instrument analysis flowchart.

The flowchart of this study for analyzing policy texts is displayed in Figure 1. First, health industry policy texts are collected automatically using crawler technology; second, policy semantic units are segmented through pre-processing of policy texts, and then, the parsed policy semantic units are classified into policy tools by utilizing the BERT deep learning algorithm; finally, the policy instruments were analyzed in multiple dimensions, and the themes of strategic initiatives instruments were identified through an LDA model with extracted keywords.

### 3.1 Establishing semantic units

This study applies the last level of subheadings as the segmentation method of semantic units considering policy text is composed in a more standardized manner, typically has different levels of headings, and retains all levels of headings up to the last level of subheadings. Policies usually have different structures, and they might not have the same level of subheadings as their last-level subheadings; therefore, the number of levels should be summarized first, and then, the last-level subheadings with their own policies text should be chosen. Additionally, policy information under the same heading has a more similar meaning to convey; therefore, the headings of each level are retained to ensure the continuity of information. The meaning to be expressed by the policy can be understood more rapidly by combining the headings at different levels to reinforce the model’s comprehension of the context.

### 3.2 Model settings

This study applies the BERT model to encode the policy text and automatically classify the policy instrument categorization. The BERT model is built based on the Transformer model, which is divided into two parts, encoder and decoder. The basic architecture of the BERT model is built on the Transformer’s encoder component, and it is a multilayer and bidirectional encoder design. The encoder part of the Transformer’s algorithm maps the natural language sequence to the hidden layer, and the decoder part maps the hidden layer to the natural language sequence. The encoder part consists of positional encoding, multi-head attention mechanism, residual linking, and layer regularization. The positional encoding mechanism determines the positional information of each word to identify the sequential relationships in the language. The Transformer model can operate in parallel in comparison to other neural network models, such as LSTM, which requires sequential iteration, significantly boosting computational efficiency. The weights of each word in a sentence are determined by performing three linear transformations on the vectors of all the words in a sentence using three matrices. The results are subsequently output through residual connectivity and regularization of the self-attentive mechanism.

The BERT model contains pre-training and fine-tuning. There are two unsupervised tasks in the BERT pre-training model, which incorporate the language tasks of random masking (masked language model) and next sentence prediction (next sentence prediction) to the multilayer bidirectional transformer encoder-based architecture. In the random masked language task, any word is randomly hidden or substituted, and the model is permitted to predict based on context, which has the advantage that the model does not rely too much on the current word at the time of encoding, but corrects errors by context. In the task of next sentence prediction, the special token [CLS] and [SEP] will be employed as the identifier for the previous and next sentences to separate them, which is convenient to enable the model to locate the position of the sentences. The subheading in the policy text supports understanding the policy meaning to identify each semantic unit and to classify the policy instruments by applying context. For instance, if the subheading is a policy objective, the subsequent text should be categorized as a policy tool in the goal planning category. The parameters can be modified in the fine-tuning process according to the corpus of policy texts.

The structure of the BERT model is as follows:

(1) Embedding. BERT embedding contains token embeddings, segment embeddings, and position embeddings. The special token [CLS] is constantly positioned at the beginning of the sentence, and [SEP] is positioned at the end of sentences to differentiate the sentences. The segment embedding corresponding to each token in sentence A is $E_{A}$ and that in sentence B is $E_{B}$ , if there are two input sentences A and B, which are represented by the symbols $E_{A}$ and $E_{B}$ as the segment embedding, respectively. Then, position embeddings can provide information about the order of words in a sentence. Finally, the input of [CLS] should consist of $E_{CLS}$ , $E_{A}$ , and $E_{0}$ .
(2) Multi-head attention. The word vectors that are obtained from embedding are linearly transformed, and the results are packed together into the query matrix, key matrix, and value matrix. Each “head” of the multiheaded attention mechanism represents a scaled dot-product attention, which is computed by the dot products of the query with all keys in a softmax function multiply the value matrix. The multi-head attention mechanism’s final output is the merging of each head’s outputs.
(3) Feedforward. The feedforward network is composed of two linear transformations with an activation in between.

The multi-label classification model is a machine learning model that can assign multiple labels to a data point. The BERT model is utilized in the multi-label classification of policy instruments, and binary cross entropy loss is employed in the model for the multi-label classification task. In the classification layer, we apply the Sigmoid function to predict the multi-label of each sample, and 0.5 is set to be the threshold value for judging the multi-label classification. The RoBERTa model and MacBERT model have been tried, which were built by BERT. The RoBERTa model robustly optimizes the BERT model in dynamic masking and replacing next sentence prediction with full-sentence task (26); in addition, whole word masking (WWM) could be combined with RoBERTa to address the Chinese word specifically. The MacBERT model adopted the masked language model (MLM) as a correction to reduce the discrepancy between pre-training and fine-tuning (27).

### 3.3 LDA topic model

The LDA topic model is a document topic generation model, proposed by Blei et al. (28), which contains three structures: document, topic, and word. The distribution of documents with subjects and topics with words constitutes a representation of the complete text. Since the learning and inference difficulties associated with the LDA model cannot be directly solved, Gibbs sampling is utilized to estimate the parameters. In the theme evolution analysis, the LDA model is applied to analyze topic changes in the strategic initiatives instruments and the topic words with high weight.

The generative process of the LDA model in a given corpus is as follows:

1. Choose $\varphi_{k}\sim Dir\left(\beta \right)$ as the word distribution $p\left(w|z_{k}\right)$ of the topic 1≤k≤K.
2. Choose $\theta_{m}\sim Dir\left(\alpha \right)$ as the topic distribution $p\left(z|w_{m}\right)$ of the document 1≤m≤M.
3. For each of the *N* words in *M* documents $1\leq n\leq N_{m}:$

- Choose a topic $z_{mn}\sim$ $\mathrm{Multinomial}\left(\theta_{m}\right)$ .
- Choose a word $w_{mn}\sim$ $\mathrm{Multinomial}\left(\varphi_{z_{mn}}\right)$ .

The parameters α and β are given, and assuming the topic number *K* is known. The text generation process based on the LDA model is Formula (1):


(1) $$\begin{array}{l} p\left(w,z,\theta ,\varphi |\alpha ,\beta \right)=\\ \overset{K}{\underset{k=1}{\prod }}p\left(\varphi_{k}|\beta \right)\overset{M}{\underset{m=1}{\prod }}p\left(\theta_{m}|\alpha \right)\overset{N_{m}}{\underset{n=1}{\prod }}p\left(z_{mn}|\theta_{m}\right)p\left(w_{mn}|z_{mn},\varphi \right) \end{array}$$

Perplexity is usually used as an indicator to evaluate the effect of the model by Formula (2). A smaller perplexity means that the model has a better predictive ability for new datasets. The calculation method is as follows:


(2) $$\mathrm{Perplexity}\;\left(D\right)=\exp\left\{-\frac{\sum_{m=1}^{M}\log p\left(w_{m}\right)}{\sum_{m=1}^{M}N_{m}}\right\}$$

## 4 Data and process

### 4.1 Data collection

The data on the health industry policy came from the official websites of the provincial governments and the Beida law treasure platform which is a database managed by the School of Law of Peking University. The keywords, such as medical care, fitness, pollution, health insurance, medicine, doctor, medical education, medical-education collaboration, nutrition, healthy aging, drug safety, and health industry, that are applied to the search are extracted from “Statistical Classification of the Health Industry (2019)” that is introduced by National Bureau of Statistics. Employing a crawler to collect policy texts before February 2023, and filtering out policy texts with the Office of State Council or provincial government office, the health industry policies were collected from 31 provinces, autonomous regions, and municipalities across the country based on data availability. Policy texts related to the epidemic were removed since the epidemic policies are relatively special, and there were 3,226 policy texts in total.

### 4.2 Policy text processing

To improve data quality and provide structured data for subsequent manual labeling and model prediction, it is indispensable to unify the format of the collected health industry policy texts, which mainly includes the following:

1. In the crawling process, most of the policy texts have structured formats; however, some policies do not have paragraph marks by parsing the texts from different attachment files and websites. Therefore, regular expressions are used to locate titles at all levels, such as chapter one, section one, and first-class subject, to perform paragraph marking and segmentation to standardize the text structure. Considering that after coding the policy texts, the connections between the contexts would be weak, which is not conducive to text recognition, and the automatic labeling is carried out in the form of subtitles at all levels, that is, each policy statement is preceded by subtitles of policies at all levels, totaling 78,343 policy statements.
2. Due to the non-standard format of the policies’ appendix, it is likely to cause misplacement when segmenting titles at all levels, so that the policy texts cannot be trained by the model and the classification results might be interfered with. Therefore, appendixes, schedules, annexes with company names, factory names, and place names in the policy texts are eliminated, and appendixes and annexes on specific policy initiatives are retained as the policy texts.

### 4.3 Trainset encoding

Rothwell and Zegveld’s method of classifying policy tools is applied to categorize them into supply, demand, and environment tools because this method could be regarded as more suitable in an industrialization background than other methods that consider government action or behavioral characteristics. The supply policy instruments provide funds, talented human resources, and scientific and technological support to the development of the health industry by the government, which can promote the development of the industry directly; the demand policy instruments are meant to drive the development of the industry by pulling the market demand; the environment policy instruments are intended to indirectly impact the development of the health industry by creating the conducive policy environment. These three types are further divided into 13 subcategories following the characteristics of health industry policies, as shown in Table 1. The category “Others” is established, totaling 14 categories, because some of the words in policy texts, including policy background or notices, do not involve specific policy initiatives. In total, 10% of the policy texts were randomly selected for manual labeling, and 8,362 labeled policy statements were acquired. We randomly select 500 policy texts from the labeled dataset to examine the consistency of the sample, and the consistency is 95.4% after relabeling the policy texts. The training set, test set, and verification set are then divided into 8:1:1 to build the model.

**Table 1:** Health industry policy instrument citation and interpretation.

Instrument types	Instrument subtypes	The interpretation of instrument types
Supply	Technology support	The government provides support for various scientific innovations and the application of internet technologies, such as improving database systems and encouraging the development of medicines and medical devices
	Fund support	Government supports the development of the health industry and increased financial investment, such as direct government investment
	Talent support	Strengthen the training of talents and improve the education and training system, such as the training of general practitioners and the introduction of talents
	Infrastructure support	Enhance the investment in infrastructure construction, improve infrastructure configuration by building hospitals and laboratories, introducing advanced equipment, and creating campus bases
	Public service	Measures formulated to enhance public services for the health industry and provide corresponding supporting public services, such as improving the service system and strengthening publicity and guidance
Environment	Goal planning	The government sets specific goals and plans to be achieved
	Regulations	Ensure an organized healthcare sector by enacting laws and regulations
	Financial support	Support companies such as concessions, tax breaks, financing, loans, and other means of financial deregulation to facilitate the development of the health industry
	Strategic Initiatives	The government formulates industrial layout and adjustment and forward-looking industrial planning to cultivate industrial growth points
Demand	Public purchasing	Utilize financial funds to purchase service products from third parties, including enterprises and non-profit organizations
	Demonstration projects	Conducting pilot projects and supporting leading enterprises
	Market shaping	The introduction of third-party forces to increase market dynamics, as well as the use of market-based approaches to price adjustments, such as the introduction of social forces to run hospitals, regulate drug prices, and cooperation with overseas institutions
	Fiscal subsidy	Subsidize the purchase of health service products by the general public to drive consumer and business demand for health
Others	Others	Other policy content that is not related to the policy tool, such as background information

### 4.4 Model parameter setting

This study implements the PyTorch structural framework for model building. Other parameters of BERT are fine-tuned as model parameters. The maximum text length is set to 512, the maximum number of characters that BERT can process, with truncating the portions of policy statements larger than 512 characters because the proportion of policy statements exceeding 512 words is relatively small, which could maintain the main semantics, as shown in Table 2. Batch_size is set to 12, utilizing AdamW as the model optimizer, and the learning rate and weight decay rate are 1e−5 and 1e−4, respectively. The number of model training iterations is set to 5.

**Table 2:** Percentage of length interval in policy statements.

Length interval	Word count	Percentage	Cumulative percentage
0–49	1,347	1.7%	1.7%
50–99	10,837	13.8%	15.6%
100–149	12,800	16.3%	31.9%
150–199	13,580	17.3%	49.2%
200–249	11,207	14.3%	63.5%
250–299	8,238	10.5%	74.0%
300–349	5,870	7.5%	81.5%
350–399	3,919	5.0%	86.5%
400–449	3,285	4.2%	90.7%
450–500	1,679	2.1%	92.9%
>500	5,581	7.1%	100.0%

Data were obtained from the authors’ calculations.

We select BERT and RoBERTa to compare the effect of the model and apply binary cross entropy loss as the loss function (BCE Loss). $y_{i}$ is the binary label of the i ’s sample, and $p\left(y_{i}=1\right)$ is the prediction of the i ’s sample by the model. The BCE Loss function is Formula (3):


(3) $$BCE\;\mathrm{Loss}=-\frac{1}{n}\overset{n}{\underset{i=1}{\sum }}\left[y_{i}*\log p\left(y_{i}=1\right)+\left(1-y_{i}\right)\log\left(1-p\left(y_{i}=1\right)\right)\right]$$

We also try the Focal Loss as the loss function (29) to increase the weight of samples with a smaller proportion. The form without α would be selected in multi-classification, γ is set to 2, and the expression of the Focal Loss Formula (4) is as follows:


(4) $$FL\left(P_{t}\right)=-{\left(1-P_{t}\right)}^{\gamma }\log\left(P_{t}\right)$$

## 5 Results and analysis

### 5.1 Research results

This study employs precision, recall, and *F*1 as the evaluation metrics for the model performance. In the multi-label classification, the recall rate can assess the proportion of the correctness predicted by the model in the actual samples, while the precision rate can assess the proportion of the correctness predicted by the model in the predicted samples. However, there would be sample imbalance in the multi-label classification of policy instruments, and the demand policy instruments have relatively few subcategories from the policy instrument distribution. The *F*1-value could give average weights to the proportion of precision and recall to reduce the negative effect of an unbalanced sample.

The BERT model has a better performance in the multi-label classification of policy instruments (30). The BERT model is finally applied after comparing BERT with other related BERT models, such as RoBERTa and MacBERT, as shown in Table 3, and the pre-training model of RoBERTa and MacBERT is Chinese-roberta-wwm-ext and Chinese-macbert-base from HFL. The performance of the Focal Loss function is only a little higher than the BCE Loss function in the *F*1-value to process an imbalanced sample problem. Therefore, this study continues to employ Bert-base-Chinese as the pre-training model and Focal Loss as the loss function of BERT, and the *F*1-value achieves 79.5%. The precision, recall, and *F*1 in each instrument type are displayed in Table 4.

**Table 3:** Comparative experimental results.

Model	Loss	Precision	Recall	*F*1
BERT	BCE Loss	0.7929	0.7885	0.785
	Focal Loss	0.7979	0.8057	0.795
RoBERTa_wwm	BCE Loss	0.8035	0.7879	0.7885
	Focal Loss	0.7685	0.8107	0.7821
MacBERT	BCE Loss	0.7943	0.7786	0.7793
	Focal Loss	0.7693	0.805	0.7807

Data were obtained from the authors’ calculations.

**Table 4:** Precision, recall, and *F*1 in each instrument type by the BERT model with Focal Loss.

Instrument types	Precision	Recall	*F*1
Technology support	0.75	0.79	0.77
Fund support	0.76	1	0.86
Talent support	0.81	0.86	0.83
Infrastructure support	0.75	0.72	0.73
Public service	0.9	0.67	0.77
Goal planning	0.92	0.77	0.84
Regulations	0.81	0.77	0.79
Financial support	0.63	0.75	0.68
Strategic initiatives	0.97	0.7	0.81
Public purchasing	0.67	0.78	0.72
Demonstration projects	0.81	0.84	0.83
Market shaping	0.58	0.8	0.68
Fiscal subsidy	0.82	0.88	0.85
Others	0.99	0.95	0.97
Average	0.7978	0.8057	0.795

Data were obtained from the authors’ calculations.

### 5.2 Evolution analysis of policy instruments

**Figure 2:**
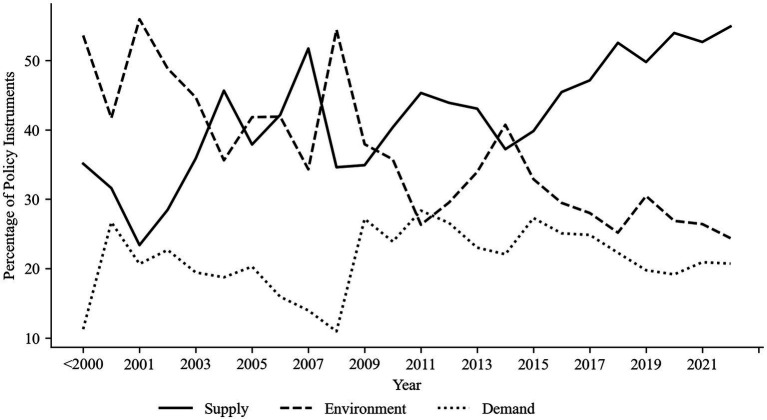
Trends of each type of health industry policy instrument.

The quantity of health industry policies released varies from year to year; therefore, the proportion of each type of policy instrument is applied to illustrate the changes in health industry policy instruments in China. The proportion of supply-side instruments gradually rises, the proportion of environmental instruments steadily declines, and after 2015, the proportion of supply-side policy instruments exceeds that of environment side, indicating that governments have gradually shifted their policy priorities for the development of the health industry from regulating the environment for industrial development to promoting industrial development, displayed in Figure 2. The demand-side policy instruments fluctuate, but they generally decline. At the same time, it also demonstrates that governments tend to employ supply-side and environment-side policy instruments in formulating policies for the health industry, and the demand-side policy instruments are fewer in quantity.

Table 5 displays the evolution and proportion analysis of several types of policy instrument subcategories, which could be adopted to further investigate the variations in policy instruments. The Chinese five-year plan represents the future policy priorities and development plans of various industries; thus, the period should be in line with it. Before 2001, the proportion of market-shaping tools on demand was relatively high, mainly because each province successively overhauled its urban medical and health systems by supporting market-driven adjustments to medical service prices and strengthening drug price management, and then, the percentage of market-shaping tools declined due to the lack of relevant policies. After 2010, it subsequently increased primarily to facilitate the accession of social capital, loosen market restrictions, and accelerate market expansion. The proportion of fiscal subsidy tools has steadily declined over time, particularly after 2015. The main reason is that, before 2005, the policy concentration was on enhancing health insurance coverage for people’s fundamental medical security. With the incremental enhancement of the medical security system, the proportion of fiscal subsidy tools has gradually decreased. Public purchasing tools have not previously taken up an enormous percentage to vary the current tendency of the demand-side instruments. Demonstration project tools have increased slightly, indicating that the government is continually implementing pilot policy innovations, but it still cannot transform the overall trend of demand-side instruments due to its low share. The proportion of supply policy instruments gradually increased above that of environmental policy instruments from 2006 to 2010, with 2009 serving as the turning point. Public service tools account for a larger proportion and display a growing trend, while regulation tools are gradually reduced, so supply instruments are more prevalent than environmental policy instruments. The rising proportion of goal planning and strategic initiatives among environmental policy instruments illustrates that the government is constantly enhancing and adjusting the planning and industrial layout of the health industry, which proves that the health industry has tremendous potential for development in the future. Financial support tools are gradually improved, which is conducive to the development of enterprises and provides a more favorable environment for enterprises in terms of tax incentives, financial credit, and financing. The percentage of infrastructure support tools in supply policy instruments continued to rise throughout the five-year plan from 2006 to 2010, and talent support tools would gradually increase in the following five-year plan. During this period, the country began to focus on the impact of technology and information on industrial development support.

**Table 5:** Percentage of the column of policy instruments subcategories (%).

Instrument types	<2001	2001–2005	2006–2010	2011–2015	2016–2020	>2021
**Supply**	**33.76**	**33.21**	**40.73**	**41.64**	**48.77**	**53.44**
Public service	27.73	24.87	26.15	20.63	23.21	21.64
Infrastructure support	1.95	3.77	5.76	7.78	7.98	12.04
Technology support	1.00	1.29	2.45	4.10	7.56	8.21
Talent support	1.73	1.24	4.08	7.26	7.90	9.80
Fund support	1.34	2.04	2.28	1.86	2.11	1.76
**Environment**	**49.00**	**46.38**	**39.89**	**32.48**	**28.12**	**25.71**
Strategic initiatives	0.84	1.15	1.82	2.68	3.40	2.40
Regulations	35.04	34.26	24.19	15.00	12.14	11.74
Financial support	4.63	3.07	2.91	3.58	3.29	4.03
Goal planning	8.48	7.90	10.97	11.22	9.29	7.53
**Demand**	**17.24**	**20.41**	**19.38**	**25.88**	**23.11**	**20.85**
Fiscal subsidy	10.21	12.96	10.53	8.68	3.86	4.16
Demonstration projects	1.67	3.30	4.15	5.72	6.97	7.07
Market shaping	4.69	3.73	3.21	8.40	10.25	7.77
Public purchasing	0.67	0.42	1.49	3.08	2.03	1.85

Data were obtained from the authors’ calculations. The bold values are first level class, and the others are the second level class.

### 5.3 Evolution analysis of provincial policy tools

The quantities of various types of policy tools utilized are slightly different, but the proportion of policy tools employed in each province is generally similar. Considering that there would be enormous dimensions by adding provinces and years, the release of health industry policies has gradually increased after 2010, and the policy system of each province is relatively complete; therefore, the “Five-Year Plan” was applied as the boundary and the provinces are divided into four major economic regions, namely, the eastern, central, western, and northeastern regions, which could helpfully analyze the proportion of policy tools used in the four major regions from 2011 to 2023 and conduct in-depth analysis on typical regions, such as the Beijing-Tianjin-Hebei region, the Yangtze River Delta, and the southeast coast, detecting the similarities and discrepancies in the health industry policy distribution.

In the health industry-related policies issued by the State Council from 2011 to 2015, supply policy tools accounted for 32.04%, environmental tools accounted for 42.04%, and demand tools accounted for 25.92%. The demand-side tool percentage of the four major economic regions is close to that of the central government, the supply-side tool percentage of the four major economic regions is higher than central government, and the environment-side tool percentage of the four major economic regions is lower than the central government, as displayed in Table 6, which illustrates provincial government focus on motivating health industry directly. From the composition of policy instruments in the Beijing-Tianjin-Hebei region, Table 7 displays that Hebei province has a higher proportion of supply-side tools and a relatively lower proportion of environment tools, and Hebei has stronger policy support which presents a rapid development of industry, especially in talent support. The policy similarity between Beijing and Tianjin is relatively high. Beijing, as the technology and innovation center in China, has paid more attention to science and technology, and the proportion of technology tools is 6.64%, which is higher than Hebei and Tianjin. In the Yangtze River Delta region, Jiangsu and Zhejiang have resembled the distribution of policy tools, while Shanghai displays certain distinctions, with a lower share of supply tools and a higher share of environment tools. The Southeast coastal region, mainly including Fujian, Guangdong, and Hainan, has a roughly similar distribution of policy instruments between each other.

**Table 6:** Percentage of policy instruments’ column in four major economic regions (%).

Time interval	Instrument types	Northeastern	Eastern	Western	Central	State Council
2011–2015	Supply	44.02	42.74	41.56	40.95	32.04
	Environment	29.62	32.76	33.09	30.14	42.04
	Demand	26.36	24.50	25.35	28.91	25.92
2016–2020	Supply	51.15	47.22	49.33	50.20	42.15
	Environment	25.90	29.69	26.82	27.93	33.41
	Demand	22.95	23.09	23.84	21.87	24.44
After 2020	Supply	56.84	55.51	53.42	51.01	35.83
	Environment	25.42	23.25	25.04	28.72	46.06
	Demand	17.73	21.24	21.55	20.27	18.11

Data were obtained from the authors’ calculations.

**Table 7:** Percentage of policy instruments’ columns in typical regions from 2011 to 2023 (%).

Time interval	Instrument types	Yangtze River Delta			Southeast coastal area			Beijing-Tianjin-Hebei		
		Jiangsu	Zhejiang	Shanghai	Fujian	Guangdong	Hainan	Hebei	Beijing	Tianjin
2011–2015	Supply	44.58	38.82	42.44	43.84	44.80	45.58	42.82	37.64	40.19
	Environment	28.32	37.76	39.53	32.27	31.52	33.88	22.36	33.95	36.14
	Demand	27.10	23.41	18.02	23.89	23.68	20.54	34.82	28.41	23.68
2016–2020	Supply	47.12	52.39	50.22	43.11	49.09	42.37	50.48	46.64	44.43
	Environment	27.25	24.44	27.36	28.86	22.38	36.77	25.52	35.78	33.99
	Demand	25.63	23.17	22.42	28.03	28.53	20.86	24.01	17.58	21.58
After 2020	Supply	51.41	62.50	54.63	58.16	51.96	57.51	59.81	51.37	58.24
	Environment	24.73	23.21	22.45	12.77	26.26	20.47	22.12	28.63	27.65
	Demand	23.85	14.29	22.92	29.08	21.79	22.02	18.08	20.00	14.12

Data were obtained from the authors’ calculations.

The supply policy tools accounted for 42.15%, environment tools accounted for 33.41%, and demand tools accounted for 24.44% from 2016 to 2020, issued by the State Council. There would be an increased proportion of supply policy tools and a decreased proportion of environment policy tools compared to the last five-year plan. In the four economic regions, the proportion of supply policy tools has been rising slightly in general. Hebei has partially stimulated its investment in infrastructure and technology in the Beijing-Tianjin-Hebei region. Jiangsu sustains a higher proportion of fund support in the Yangtze River Delta region. Guangdong has boosted the proportion of supply policy tools and begun to raise the proportion of talent support policy tools; furthermore, Fujian has paid more attention to the demand policies to build a conducive market environment for enterprise in the Southeast coastal region.

Central government relatively focuses on environmental policy instruments after 2021, even though the quantity of policies is small due to the unterminated five-year plan; however, other provinces prefer to supply policy tools. Hebei has seen greater growth in infrastructure development and technology, while Beijing still places emphasis on science and technology support, and Tianjin is prominent in the financial subsidy category. In the Yangtze River Delta region, Zhejiang has a significant increase in the proportion of talent support, resulting in an increased percentage of supply policy instruments. In the Southeast Coast region, Guangdong’s proportion of supply policies declined, but talent support policies remained relatively high.

### 5.4 Evolution analysis of policy tools’ topic

#### 5.4.1 Topic analysis of policy classification

The health industry contains more subcategories, and categorizing the policies can more clearly determine the proportion of policy instruments implemented and dissect the development direction of the health industry. Due to the large number of policy-searching words, classifying the policy-searching words in the policy collection can reduce the analysis dimension. There are nine topics acquired, which are insurance, health industry, rehabilitation, sports, pollution, medical services, medical education, medicine, and nutrition. The 16 keywords from the title of policies are discovered and extracted to complement the topic classification after using search words as topic categories when we read and label the training samples of policy texts manually. The method of topic classification is shown in Table 8. From 2011 to 2015, the percentage of pollution policy tools gradually fell, illustrating that environmental problems had begun to ameliorate and that the policy’s attention was beginning to shift in other directions. Medical and pharmaceutical policies remained the top priority of health industry policies. The proportion of sports and fitness policies gradually grew in 2020, illustrating that China’s health industry gradually began to move from medical to health maintenance, sports, and commercial health insurance. From the subcategories of policy instruments in Table 9, the balanced expansion further indicates that the share of the health services industry is gradually rising.

**Table 8:** Method of topic classification by search words and keywords of policies’ titles.

Topic	Search words	Keywords of policies’ title
Insurance	Health insurance	Insurance
Health industry	Health industry	Health
Health maintenance	Healthy ageing	Older individuals’ care
		Health maintenance
		Tourism
Sports	Fitness	Sports
Nutrition	Nutrition	Food safety
		Reasonable diet
Pollution	Pollution	
Medical services	Doctor	Social medical care
	Medical care	Hospitals
		Chronic diseases
		Hygiene
		Medical care
Medical education	Medical education	
	Medical-education collaboration	
Medicine	Medicine	Essential medicines
	Drug safety	Drugs
		Generic drugs

**Table 9:** Percentage of rows of policy instruments in each topic (%).

Time interval	Instrument types	Insurance	Health industry	Health maintenance	Sports	Pollution	Medical services	Medical education	Medicine	Nutrition
2011–2015	Supply	2.53	2.53	0.50	4.96	29.71	45.46	0.00	9.90	4.42
	Environment	5.84	2.38	0.77	2.80	44.42	32.08	0.00	8.45	3.28
	Demand	3.62	4.11	0.45	1.30	17.06	63.46	0.00	7.11	2.89
2016–2020	Supply	4.21	14.28	0.80	7.19	17.60	35.58	3.78	13.17	3.39
	Environment	7.22	10.81	1.25	8.14	26.76	29.82	0.56	13.07	2.36
	Demand	3.84	14.78	1.02	12.97	10.41	41.15	1.97	11.54	2.30
After 2020	Supply	9.01	1.34	3.90	13.65	7.85	33.01	9.87	20.65	0.72
	Environment	18.09	2.21	4.58	7.43	13.36	32.57	1.58	18.84	1.35
	Demand	10.87	2.96	5.46	14.95	5.51	32.86	6.20	21.15	0.05

Data were obtained from the authors’ calculations.

It is evident that, in the medical service theme, the proportion of fiscal subsidy tools in demand-side policy instruments gradually decreased from 26.48% to 5.88% between 2011 and 2023, and the proportion of infrastructure tools in supply-side policy instruments gradually increased from 5% to 8.09%, and the proportion of technology support also grew from 4.57% to 5.05% at the same time. The basic medical insurance system was not impeccable in the early stage, and the government’s fiscal support was indispensable to promote it. Then, infrastructure and technology research and development turned out to be the focused investment target with the improvement of the medical insurance system. In the medicine theme, the percentage of technological support grew from 1.23% to 4.56% between 2011 and 2023, and the proportion of market tools in demand-side policy instruments gradually increased from 2.93% to 10.55% at the same time. Since pharmaceutical innovation plays an essential part in disease control, an open research environment is indispensable for attracting third-party power and achieving continuous innovation, which could stimulate market demand and expand the scale of the health industry.

#### 5.4.2 Evolution analysis of policy texts’ topics

**Figure 3:**
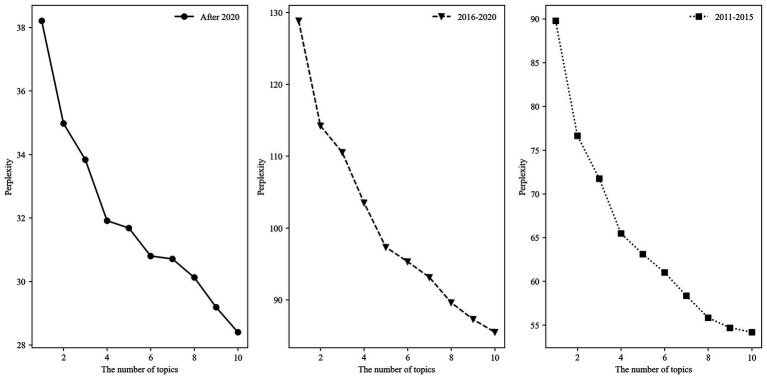
Perplexity trends of strategic initiatives instruments under different topic numbers in each period.

The development of the health industry is inseparable from policy support. In 2013, the State Council issued “Several Opinions of the State Council on Promoting the Development of Health Service Industry,” releasing the signal to foster the mainly related industries for supporting the health service industry and to support the development of diversified health services. Subsequently, the central and provincial governments have begun releasing planning policies for the health industry and health service industry. The strategic initiatives are primarily based on government planning and the industrial layout of the industry; therefore, they are screened as the primary analysis target in the theme analysis of the policy tool. Building a health industry dictionary is indispensable, which has been expanded with proper nouns, such as medical association, general practitioner, and fitness circle, to combine with Jieba subscripts, and then it has been employed as the tool to cut the policy text. The segmented policy text is vectorized by word frequency matrix representation, and the lda package in Python is employed to construct an LDA topic model. The perplexity trend figure’s turning point indicates that 4, 5, and 4 are the optimal number of topics for each period. Figure 3 displays this by generating some of the top 10 keywords under each topic to investigate the changes in strategic initiatives focused on the “health industry” topic among the five-year plans.

**Figure 4:**
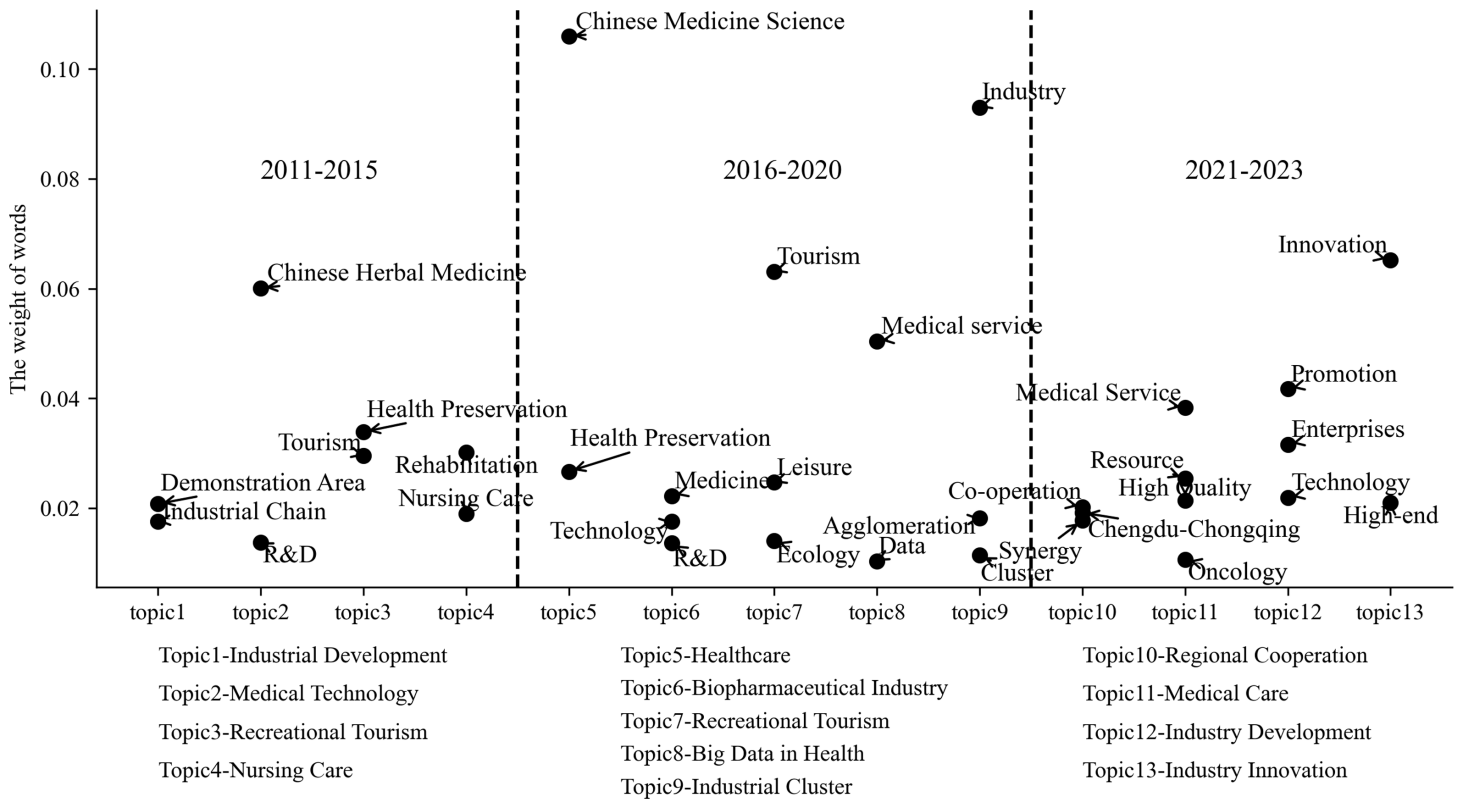
Topic change of health industry strategic initiatives based on LDA topic words’ weight.

The policies that are related to industrial planning apply more strategic initiatives. In 2013, the State Council issued “Several Opinions of the State Council on Promoting the Development of Health Service Industry” to accelerate the development of diversified health services such as healthy older individuals services, Chinese medicine, and healthcare services. The “General Office of Zhejiang Provincial People’s Government on Opinions on Accelerating the Development of Chinese Medicine Health Services” document, published by Zhejiang Province in 2015, emphasized the criticality of developing the Chinese medicine healthcare industry in addition to the health tourism and Chinese medicine equipment industries. As indicated in Figure 4, the themes of the strategic initiatives are primarily concerned with “Chinese medicine,” “rehabilitation,” and “nursing care,” etc. by choosing the higher weight of words, which may represent the policy tendency from 2011 to 2015. In 2016–2020, the weight of keywords, such as “agglomeration,” “clustering,” and “data,” increased, and the main reason is that in 2016, the State Council issued the “General Office of the State Council on promoting and regulating the development of healthcare big data application guidance” to comprehensively deepen the application of healthcare with big data in various aspects of healthcare industry governance, medical clinical, scientific research innovation, public health, medical intelligent equipment, etc. The State Council published the Opinions of the General Office of the State Council on Promoting the Development of “Internet Medical Health” in 2018. This document involves telemedicine services, health management, medication management, doctor appointments and consultations, and medical insurance payment, among other topics. It will incorporate Internet technology in numerous ways to accomplish interconnection, boost the process of the medical association, enhance the efficiency of medical services by integrating Internet medical resources, and achieve effective business collaboration. On the other hand, the prominence of industrial innovation additionally keeps gradually expanding, relying on industrial centers and enterprise resources to promote the agglomeration of biomedical industries, support enterprise innovation, such as research and development in biopharmaceuticals and other domains, and improve the industrial chain. From 2020 onward, keywords such as “innovation,” “high-end,” and “chengdu-chongqing” have more significance in the theme of industrial transformation and industrial innovation. It demonstrates that the integration of industry within the region and the development of industrial refinement have been given more emphasis, and the development of the health industry would be guided to become high-end through industry innovation, which also confirms the direction of the Chinese economy’s high-quality development from another perspective.

## 6 Discussion

In this study, the deep learning algorithm is linked to the multi-label classification of policy instruments, which enhances the classification precision of policy instruments, and the average precision is nearly 80%, after the addition of the Focal Loss function. Moreover, enlarging the research sample to explore the evolution analysis in multiple dimensions could be possible by knowing the research results. In some traditional research methods, manual coding is relatively subjective, time-consuming, and hard to replicate, especially, for very large datasets. The deep learning method could improve the efficiency of the policy analysis. Some study proves that combining rigorous manual labeling methods with machine learning approaches could offer a good performance to reduce the time cost (31). In addition, some study also demonstrates that there is a good performance in less classification and short sentences (32). In policy analysis, due to the different data sources, sentences and paragraphs are often used as analysis units in most policy texts, but some studies are not (33). The last-level subheadings for establishing semantic units are applied, which can reinforce the coherence of context, and process the large datasets with uniform specifications automatically.

Qualitative analysis and content analysis are often applied to policy instrument studies by manually coding the policy text. However, some research just explores the industry plan of each province, which involves 19 provinces and 57 policy texts (34). This study presents an application of deep learning in health industry policy instruments based on the characteristics of the policy texts. Applying the multi-label classification method could provide a more detailed class and detect more information than the multi-class classification method in long paragraphs. Considering the unbalanced sample of policy instruments, this study combines the Focal Loss function to increase the weights of smaller samples and detects the rationality of the policy instruments’ collocation to increase the rationality of the model. There is not enough sample in the whole industry policy, and comparing the policy instruments in sub-sectors could be difficult with a small policy dataset. Therefore, expanding the study sample might be more tangible for comparing the instrument distribution among provinces and topics. As shown in Tables 7, 8, some key regions’ governments prefer the policy instruments mix that is suitable for local development, but there are still some similarities among the policy instruments mix of the four major economic regions. The topic of medical service and medicine has nearly 50% in the health industry, which is not realized by the small samples. The BERT multi-label method enlarges the sample dataset in the health industry that involves many industry sectors. The monitoring system of policy instruments should be in a long-term and efficient way, and applying a relatively comprehensive dataset of policy text is beneficial to the development of China’s health industry.

## 7 Conclusion

This study proposes a framework for classifying health industry policy instruments and analyzing the evolution of the policy instruments. First, the BERT model with Focal Loss is employed for policy instruments’ multi-label classification, which enhances the accuracy and provides a boost to analyze the policy instruments more efficiently.

Second, in the temporal dimension, the proportion of supply policy instruments has increased since 2008, the proportion of environment policy instruments has gradually decreased, and the proportion of demand policy instruments has fluctuated and has slightly declined. The supply and environment policy instruments are predominant, while the demand instruments are neglected relatively. The government is paying more attention to the health industry by offering many direct supports to accelerate industry development, and marketization still needs to be improved. Regulations and public service have a large percentage, which illustrates the environment of the health industry has been gradually normalized; however, the low percentage of public purchasing may affect the order volumes of pharmaceutical companies and medical institutions, which would limit the growth of health industry, and the absence of market-shaping instruments might cause unequal distribution within the health industry and discourage social capital from getting involved in the market, both of which are unfavorable to the formation of a diversified market and a healthy competitive environment.

Third, multi-label classification makes the expansion of the sample, and the composition of regional policy instruments could be compared by the model predicted result. In the spatial dimension, the proportion of policy instruments among the four major economic regions is similar, with provincial governments having a higher proportion of supply policy instruments than the central government and a slightly lower proportion of environment ones; among the key regions, Beijing and Tianjin have similar policy instrument combinations and an increased level of synergy, while Hebei is vigorously catching up in terms of policy support; the Yangtze River Delta region’s Jiangsu and Zhejiang have resembling distributions, while Shanghai illustrates some variations, and the southeast coastal region has similar instrument compositions. A similar mix of policy tools could make governments learn the successful experience easier, but this might leave governments with limited space for creativity and flexibility to promote local characteristic industries and confront the special challenge. Moreover, it may not be conducive to the effective allocation of resources according to the actual local situation without considering its own resource endowment and economic structure.

Finally, the three issues of healthcare, medicine, and pollution continue to be the focus of policy instruments in the thematic dimension, but the proportion of health maintenance, sports, and insurance is increasing, indicating a trend of shifting policy focus. The policy emphasizes the themes of fitness, healthcare, and health tourism between 2011 and 2015, as can be seen from the primary phrases extracted for the strategic initiatives of the health industry theme, the weight of data and information has increased with the introduction of the Internet and big data concepts during the years 2015–2020, and, after 2020, industrial development will focus a greater deal on innovation and improvement to propel the sector toward high-end development.

This framework could improve the efficiency of refining the health industry policy instruments based on the deep learning method. In addition, it can reduce the time cost of manual coding and detect more labels in the policy paragraph by applying a multi-label classification method. The health industry includes several subfields, such as medical service, health education, and pollution control, which contain plenty of policies year by year. This framework could process a very large dataset that is suitable for the complex health industry, and comparing the difference between provincial and topic dimensions could be possible. The policy text can be transferred into valuable information to help policymakers master the health industry policy instrument system.

Health policy instrument analysis can only judge the development of the provincial health industry from macro-level policy application, but it cannot evaluate the effect of a specific policy. Due to the limited space, the evolution analysis of policy instruments by province and topic is in a segregated manner. Each province has its priorities, and the order of policy instruments should be considered according to the particular situation. Cross-analysis in multi-dimension will be considered in the future. In addition, due to the different writing preferences of governments’ policy texts, different levels of policy texts would exist. Therefore, calculating the proportion of sections below each level for a better segment of each semantic unit will be considered in further study.

## Data availability statement

The original contributions presented in the study are included in the article/supplementary material, further inquiries can be directed to the corresponding author.

## Author contributions

JJ: Conceptualization, Funding acquisition, Investigation, Project administration, Supervision, Writing – review & editing. HD: Conceptualization, Data curation, Formal analysis, Investigation, Methodology, Project administration, Software, Validation, Visualization, Writing – original draft, Writing – review & editing.
